# Supplementary material for: Content-rich biological network constructed by mining PubMed abstracts
Source: BMC Bioinformatics. 2004 Oct 8;5:147. doi: 10.1186/1471-2105-5-147 (PMC528731; doi:10.1186/1471-2105-5-147)
Supplement: Additional File 5 — The original Chilibot query results of the term "long-term potentiation (LTP)" and 22 other terms, limiting the latest references analyzed to the years 1990, 1995, 2000, and 2004. [file 1471-2105-5-147-S5.bz2 › chilibotAdditionalFile5/ltp2004/html/PKC_ATF.html]

 


 **PKC** and **ATF** 
  
Found 19 abstracts in PubMed,  **19 abstracts were retrieved and analyzed**.  


---

 Search Google  |
 PDF files only 
|  EDU domain only 

---

**Interactive relationship** (e.g. stimulation, inhibition, etc)

- The phosphorylation of the serine residue at position 121 of  **ATF**  2 appears to be induced by protein kinase C alpha  **PKC**  alpha after treatment of cells with retinoic acid RA or induction with E1A.  Ref: 9436983 Genes Dev, 1998
- Furthermore, high calcium o, in a  **PKC**  independent fashion, induces phosphorylation of ERK1 2, SEK1, p38 MAPK, and its downstream transcription factor  **ATF**  2.  Ref: 12700162 Am J Physiol Endocrinol Metab, 2003
- We observed that u PA and u PA  **ATF**  stimulated chemotactic migration of both LB6 clone 19 cells and human fibroblasts, which could be impaired by down regulation of protein kinase C  **PKC**  with phorbol myristate acetate PMA.  Ref: 8050501 Exp Cell Res, 1994
- Collectively, these results indicate the DSE are TRE like cAMP responsive elements that bind both AP 1 and CREB  **ATF**  permitting induction of the HIV 1 LTR by both  **PKC**  and PKA activation signals.  Ref: 9201233 Virology, 1997

**Parallel relationship** (e.g. studied together, co-existance, homology, etc.)

- These results suggest that  **ATF**  2 and p300 cooperate in the control of transcription by forming a protein complex that is responsive to differentiation inducing signals, such as RA or E1A, and moreover, that the phosphorylation of  **ATF**  2 by  **PKC**  alpha is probably a signaling event in the pathway that leads to the transactivation of the c jun gene in F9 cells.  Ref: 9436983 Genes Dev, 1998
- Induction of TGF beta 1 synthesis by 30 mmol D glucose was associated with induction of  **PKC**  alpha, phosphorylation of cAMP responsive element binding protein CREB and activating transcription factor 1  **ATF**  1, and increased fibronectin and type I collagen translation.  Ref: 12846747 Kidney Int, 2003
